# The incretin axis offers a novel therapeutic target to preserve myocardial energy metabolism in cardiorenal syndrome

**DOI:** 10.1186/1532-429X-18-S1-O15

**Published:** 2016-01-27

**Authors:** Marie A Schroeder, Chuck Cunningham, Albert Chen, Kim A Connelly

**Affiliations:** 1 St Michael's Hospital, Toronto, ON Canada; 2 Sunnybrook Health Sciences Centre, Toronto, ON Canada

## Background

Clinical and epidemiological data have identified a cardiorenal syndrome (CRS), in which heart and/or kidney failure accelerates dysfunction in the other organ. New therapeutics are needed to target the mechanisms that cause CRS and treat the whole patient. The aims of this study were to 1) assess *in vivo* cardiorenal metabolism using hyperpolarized ^13^C MR spectroscopy (MRS) in experimental CRS, and 2) to test the hypothesis that normalizing aberrant metabolic reprogramming could provide CRS therapy.

## Methods

The diabetic Goto-Kakizaki [GK] rat, aged to 40 weeks, were used as a model of secondary CRS and compared with age matched Wistar controls. Animals underwent echocardiography at 8 weeks of age, and subsequently every 4 weeks. A cohort of paired animals (*n = 5*) underwent invasive cardiac catheterization for pressure-volume (PV) loop analysis. In a second cohort of animal pairs (*n = 4*), hyperpolarized [1-^13^C]pyruvate was infused intravenously and ^13^C MR spectroscopic data were acquired from hearts and kidneys. An interleaved pulse-acquire pulse sequence was used (1.2 cm axial slice through alternately heart or kidneys, 20° tip angle, TR=1 s). Daily treatment with glucagon-like peptide-1 (GLP-1) receptor agonist liraglutide (0.2 mg/kg) was given to a third cohort GK rats for 10 weeks (*n = 4*) prior to ^13^C MRS assessment of metabolism. Cardiac and renal tissue was collected for histopathological and molecular analysis.

## Results

**Figure 1:**
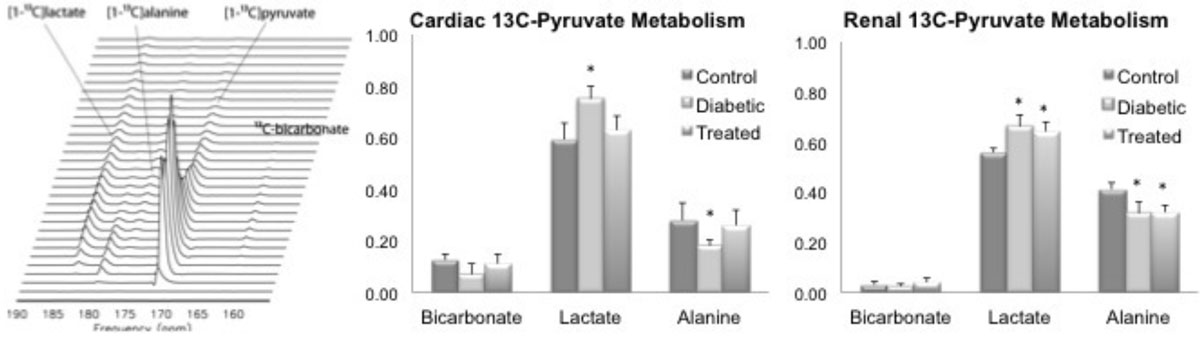
**Left Representative series of MR spectra acquired from the heart of a diabetic rat, following infusion of hyperpolarized [1-**^**13**^**C]pyruvate, and the corresponding quantification (middle and right)**. The input bolus of pyruvate is evident, as are its enzymatic conversions to other metabolites and the decay of the hyperpolarized signal. Data are expressed as ratios of each observed metabolite to the total observed ^13^C metabolicm, which did not change with diabetes or liraglutide treatment. Hyperpolarised ^13^C MRS data were obtained from the kidneys during the same [1-^13^C]pyruvate infusion, with spectra acquired in between cardiac spectra (a 1 s offset).

Glycated hemoglobin (HbA1c) confirmed that GK rats were diabetic at 20 weeks. Forty-week-old untreated GK rats developed proteinuria, LV hypertrophy, and pulmonary congestion. PV-loops demonstrated preserved systolic, yet impaired diastolic function. Histology demonstrated myocyte and glomerular hypertrophy, interstitial fibrosis and glomerulosclerosis. Hyperpolarised ^13^C MRS data indicated that cardiorenal carbohydrate metabolism was reprogrammed to promote lactate production over oxidation (Figure 1). In the kidney, ^13^C-lactate was increased at the expense of ^13^C-alanine. Metabolic reprogramming was likely mediated by inflammation (in both organs, macrophage infiltration and toll like receptor 4 protein expression were increased) or maladaptive systemic gluconeogenesis (renal *Pck1* and *G6pc* mRNA were increased). Liraglutide treatment reduced HbA1c levels in GK rats by 13%. The drug normalized carbohydrate utilization to abrogate ^13^C-lactate production in the heart (Figure 1). In the kidney, no effect of liraglutide treatment was observed.

## Conclusions

Hyperpolarized ^13^C MRS identified that in diabetes-induced CRS, whole-body carbohydrate utilization was impaired and represented a novel target for therapy. We conclude that 1) non-invasive metabolic assessment using hyperpolarized ^13^C MRS offers an important tool to investigate the pathology of multi-organ diseases, and to identify and evaluate new therapeutic approaches, and 2) that liraglutide therapy may have a role in treating diabetes-induced CRS by preserving myocardial function.

